# Supplementary material for: Realization of a crosstalk-avoided quantum network node using dual-type qubits of the same ion species
Source: Nat Commun. 2024 Jan 3;15:204. doi: 10.1038/s41467-023-44220-z (PMC10764850; doi:10.1038/s41467-023-44220-z)
Supplement: Supplementary file 1 — Supplementary Information [file 41467_2023_44220_MOESM1_ESM.pdf]

Supplementary Information for "Realization of a crosstalk-avoided quantum network node using dual-type qubits of the same ion species"

L. Feng,[1,*] Y.-Y. Huang,[1,*] Y.-K. Wu,[1,2] W.-X. Guo,[1,3] J.-Y. Ma,[1,3] H.-X. Yang,[3] L. Zhang,[1] Y. Wang,[1] C.-X. Huang,[1] C. Zhang,[1] L. Yao,[3] B.-X. Qi,[1] Y.-F. Pu,[1,2] Z.-C. Zhou,[1,2] and L.-M. Duan[1,2,4,†]

[1]*Center for Quantum Information, Institute for Interdisciplinary Information Sciences, Tsinghua University, Beijing 100084, PR China*
[2]*Hefei National Laboratory, Hefei 230088, PR China*
[3]*HYQ Co., Ltd., Beijing 100176, PR China*
[4]*New Cornerstone Science Laboratory, Beijing 100084, PR China*

SUPPLEMENTARY NOTE 1 – ERROR SOURCES OF ION-PHOTON ENTANGLEMENT

The measured ion-photon entanglement fidelity in the main text comes from multiple error sources. It is worth noting that, given the overall infidelity above 10%, estimating its individual components can be inaccurate and that different parts may not add up together directly.

As described in the main text, we achieve an entanglement generation rate of about $5\,\mathrm{s}^{-1}$ at the repetition rate of $1.6 \times 10^4\,\mathrm{s}^{-1}$. This means that we have about 3200 attempts per succeeded event. In each attempt, the PMT can collect photons in a time window of 60 ns. Given the dark count rate of about 100 Hz of the PMT, we estimate about 0.02 dark count per succeed event, thus 2% infidelity in ion-photon entanglement.

The SPAM error mainly comes from the detection infidelity of the ionic qubit state. When using a PMT to collect the photons scattered from the ion under the detection beam, we measure 1.5% error for the bright state and 0.5% error for the dark state, assuming perfect state preparation via optical pumping and microwave pulses. Plugging these errors into our bound for entanglement fidelity in the main text

$$F \geq \frac{1}{2}[P(\uparrow,V) + P(\downarrow,H) - 2\sqrt{P(\downarrow,V)P(\uparrow,H)} + P(\tilde{\uparrow},\tilde{V}) + P(\tilde{\downarrow},\tilde{H}) - P(\tilde{\uparrow},\tilde{H}) - P(\tilde{\downarrow},\tilde{V})], \quad (1)$$

we estimate 2% error due to the imperfect state detection. (Note that in this bound they add up together rather than being averaged.) Later when combining ion-photon entanglement generation and long-time memory qubit storage together, we use an EMCCD for the spatially resolved detection of the two ions. Without additional quantum enhancement, our EMCCD has lower quantum efficiency of about 23% at 370 nm than that of the PMT of about 30%. Besides, we only select the pixels near the individual ions for their detection to suppress the crosstalk, which further reduces the number of collected photons. Therefore, to maintain the separation between the photon counts for bright and dark states, we increase the detection time from 300 μs for the PMT to 1 ms for the EMCCD. This, in turn, results in larger off-resonant pumping of the bright and the dark states to decay into each other. As a consequence, the detection errors for the bright state and the dark state increase to 3% and 2%, respectively, and thus 5% error on the bound of entanglement fidelity.

Deviation of the 370 nm pulsed laser from perfect π polarization also leads to imperfect ion-photon entanglement. This can be calibrated by a similar experimental sequence as Fig. 1**b** of the main text. Specifically, we initialize the ion in $|^2S_{1/2}, F=1, m_F=0\rangle$, apply a π pulse, and then measure the population in $|^2S_{1/2}, F=0, m_F=0\rangle$ after the spontaneous emission conditioned on the detection of the $|H\rangle$ or $|V\rangle$ photon. Ideally, a perfect π-polarized laser pulse will only excite to $|^2P_{1/2}, F=0, m_F=0\rangle$ such that the final state will remain in the $^2S_{1/2}, F=1$ manifold. On the other hand, the $\sigma^\pm$ polarization components can lead to excitation to the $|^2P_{1/2}, F=1, m_F=\pm1\rangle$ levels, which decay to $|^2S_{1/2}, F=0, m_F=0\rangle$ with 1/3 probability. In the experiment, after calibrating the orientation of the laser and the magnetic field, we still get about 0.4% conditional population in $|^2S_{1/2}, F=0, m_F=0\rangle$ (after subtracting the detection error of the bright state as described above). Note that it is the difference between two populations accurate to the 1% level, therefore we shall only regard it as an upper bound for the error. Suppose the excitation to the $|^2P_{1/2}, F=1, m_F=\pm1\rangle$ levels is ϵ, then we have $0.4\% = (\epsilon \times 1/3 \times 1/2)/(1/3 + 2/3 \times 1/2) = \epsilon/4$, where the factor of 1/2 represents the mapping from σ polarization to H polarization. This suggests that we have about

* These authors contribute equally to this work
† lmduan@tsinghua.edu.cn

$\epsilon = 1.6\%$ infidelity in the prepared ion-photon entangled state. In this experiment, since we are using Eq. (1) to bound the fidelity, we further consider the effect of the imperfect polarization to the obtained ion-photon correlation. Ideally, the excited state $|^2P_{1/2}, F = 0, m_F = 0\rangle$ has 1/3 probability to decay to $|^2S_{1/2}, F = 1, m_F = 0\rangle$ and to emit a π-polarized photon. Now the excitation to $|^2P_{1/2}, F = 1, m_F = \pm 1\rangle$ gives an additional probability of $\epsilon/3$ to get a π-polarized photon together with the $|^2S_{1/2}, F = 1, m_F = \pm 1\rangle$ states, which later will be converted into the dark state by the microwave pulses. In this sense, we get an error in the conditional probability $P(\downarrow |V) = (\epsilon/3)/(1/3) = \epsilon$. Similarly, ideally we have 2/3 probability to get a σ-polarized photon with a final dark state, while now due to the imperfect polarization we get additional $2\epsilon/3$ probability for a σ-polarized photon with a final bright state, i.e. $P(\uparrow |H) = (2\epsilon/3 \times 1/2)/(2/3 \times 1/2) = \epsilon$. Plugging into Eq. (1), we get an error in the fidelity bound of $2\epsilon = 3.2\%$.

Infidelity can also arise from the misalignment of the objective to collect the photon, which leads to polarization mixing of the measured π and $\sigma^\pm$ components. In the experiment, we calibrate this polarization basis by applying a continuous-wave 370 nm laser backward along the single-photon-detection path onto the ion. The laser is resonant to the transition between $|^2S_{1/2}, F = 1\rangle$ and $|^2P_{1/2}, F = 1\rangle$. Ideally when the detection path is set to π polarization, the transition is electric-dipole-forbidden for an initial $|^2S_{1/2}, F = 1, m_F = 0\rangle$ state such that it will remain bright under this laser. On the other hand, nonzero $\sigma^\pm$ components will lead to excitation to $|^2P_{1/2}, F = 1, m_F = \pm 1\rangle$, which will further decay to the dark state $|^2S_{1/2}, F = 0, m_F = 0\rangle$. By turning on this laser for a fixed time and adjusting the orientation and the polarization of the detection path, we optimize the single-photon-detection setup to give as high as possible the final bright state population. To estimate the error due to this calibration method, we observe that in the experiment the final state is not significantly influenced when the wave plates are rotated by about 5°. Therefore, an error of $5^\circ \approx 0.1$ can still exist in the polarization basis, which in turn results in an error in the ion-photon correlation as $P(\downarrow |V) \sim P(\uparrow |H) \sim 0.1^2 = 1\%$ and finally an error in the entanglement fidelity bound as 2%.

Finally, we consider the error when converting $(|^2S_{1/2}, F = 1, m_F = 1\rangle + |^2S_{1/2}, F = 1, m_F = -1\rangle)/\sqrt{2}$ into $|^2S_{1/2}, F = 0, m_F = 0\rangle$. As we describe in the main text, there is a random phase accumulation between the $|^2S_{1/2}, F = 1, m_F = \pm 1\rangle$ levels due to the uncertainty of the time when the spontaneous emission occurs. This is compensated by feedforwarding the detection time of the photon into the phase of the two-tone microwave pulse. However, there is a jitter time of about $\Delta t \approx 2$ ns from the PMT, the sequencer and the arbitrary waveform generator (AWG) applying the microwave pulse. Considering the frequency difference of $\Delta f = 2 \times 1.4\text{MHz/G} \times 5.6\text{G} = 16\,\text{MHz}$ between the two levels, we estimate a phase uncertainty of $\Delta\phi = 2\pi\Delta f \cdot \Delta t = 0.2$, or an infidelity of $\Delta\phi^2 = 4\%$. Also, we measure a dephasing time of $T_2 \approx 530\,\mu$s of the two Zeeman levels, such that an error of about $13\,\mu\text{s}/530\,\mu\text{s} \approx 2\%$ can occur during the microwave pulse due to the shift of the Zeeman levels under external magnetic field.

SUPPLEMENTARY NOTE 2 – CROSSTALK ERROR DUE TO SPONTANEOUS EMISSION OF NEARBY IONS

We can divide the crosstalk in this experiment between different ions into two classes: (1) crosstalk due to broad addressing beams which can in principle be suppressed by focusing into a narrower beam waist, and (2) crosstalk due to the random scattering of photons from ions which is inevitable in laser cooling, optical pumping, state detection, and ion-photon entanglement generation. Also note that, for infrared or microwave driving fields, it is often difficult to focus these beams to a smaller size than the ion spacing, so that we should design the experimental sequence to use them as global beams, rather than regarding them as crosstalk errors.

In this experiment, we use a global 370 nm laser and a global microwave for the initialization of the S-qubit. We have shown in a previous work that the dual-type qubit scheme can suppress their crosstalk to be below 10^{-3} [1]. On the other hand, had we encoded the memory qubit in S-states, i.e. without dual-type qubit encoding, then the state of the memory qubit would have been destroyed completely in each attempt to generate ion-photon entanglement.

Nevertheless, the above crosstalk can in principle be avoided by using a focused 370 nm laser even without the dual-type qubit encoding. Here we further consider the inevitable crosstalk error due to the spontaneous emission of other ions. We will take the ion-photon entanglement generation process as an example with one photon per entanglement generation attempt. Note that there is also photon scattering in the Doppler cooling and the optical pumping before each attempt, which will increase our calculated crosstalk error below by tens of times.

Consider an ion with average population ρ in the excited state, radiating fluorescence at the rate $\rho\Gamma$ where Γ is the spontaneous emission rate of the excited state. Each emitted photon has energy $\hbar\omega_0$. For simplicity, suppose the radiation distributes uniformly in all spatial directions. Then the radiation intensity at a distance d from the ion is given by

$$I = \frac{\hbar\omega_0}{4\pi d^2}\rho\Gamma. \tag{2}$$

On the other hand, we have saturation intensity for the resonant driving [2]

$$I_{sat} = \frac{\hbar \omega_0^3 \Gamma}{12\pi c^2}, \tag{3}$$

so that this near-resonant fluorescence will cause a Rabi frequency Ω on a nearby ion at a distance of d, satisfying

$$\Omega^2 = \frac{\Gamma^2}{2} \frac{I}{I_{sat}} = \frac{3c^2 \rho \Gamma^2}{2\omega_0^2 d^2} = \frac{3\lambda^2 \rho \Gamma^2}{8\pi^2 d^2}, \tag{4}$$

where λ is the wavelength of the fluorescence light. From this, we further get the excitation probability of the nearby ion

$$\rho' = \frac{\Omega^2}{2\Omega^2 + \Gamma^2} \approx \frac{\Omega^2}{\Gamma^2}, \tag{5}$$

namely a spontaneous emission error for time duration τ

$$\epsilon = \rho' \Gamma \tau = \frac{3\lambda^2}{8\pi^2 d^2} \rho \Gamma \tau. \tag{6}$$

As an estimation, for Doppler cooling under saturation parameter $s = 1$ and a detuning $\Delta = -\Gamma/2$, the excitation probability of the cooling ion is $\rho = 1/6$. Then the spontaneous emission error for an ion at a distance of $d = 400\,\mu$m for $\tau = 500\,$ms will be $\epsilon = 34\%$. This is comparable to the results in our previous work where the decoherence error of an edge ion in a long quasi-1D ion crystal is measured with the central ions providing sympathetic cooling [3].

In this experiment, we excite the ion at the rate of $1.6 \times 10^4\,\mathrm{s}^{-1}$, thus we replace $\rho\Gamma \to 1.6 \times 10^4\,\mathrm{s}^{-1}$. Then for the ion-photon entanglement generation time $\tau = 100\,$ms (half of the storage time), we estimate a crosstalk error $\epsilon = 6\%$ for an S-qubit at a distance of $d = 12\,\mu$m. Furthermore, as shown in Fig. 1**c** of the main text, before each ion-photon entanglement attempt, we initialize the communication ion by Doppler cooling and optical pumping. During the $40\,\mu$s Doppler cooling, hundreds of photons will be scattered, but how frequently we need to perform the Doppler cooling depends on the heating rate and thus has room for improvement. In comparison, the photon scattering during optical pumping is more intrinsic. Since the $^{171}\mathrm{Yb}^+$ ion has 1/3 probability to decay to the dark state $|\downarrow\rangle$ after each spontaneous emission, we can estimate a state preparation infidelity of about $(1 - 1/3)^k$ after the emission of k photons. In other words, about $k = 11$ photons will be necessary for a state preparation error of 1%. Therefore, we estimate an intrinsic crosstalk error of $1 - (1 - \epsilon)^{k+1} \approx 50\%$, had we encoded the memory qubit in the S state.

On the other hand, for an F-qubit, since the scattered photon is far off-resonant, the excitation probability will be reduced to $\rho' \approx \Omega^2/(4\Delta^2)$ [2]. For the $F_{7/2}$ levels, the closest transition might be at 364 nm with detuning $\Delta \sim 2\pi \times 10\,$THz and a linewidth on the order of MHz [4]. The spontaneous emission error will thus be suppressed by a factor of $\Gamma^2/(4\Delta^2) \sim 10^{-12}$, which is far below the fault-tolerant threshold and can be safely neglected. Besides, for far-detuned driving, a considerable fraction of this spontaneous emission will be Rayleigh scattering which does not lead to decoherence [5]. As we show in the main text, we measure the same storage fidelity for the F-qubit with/without the ion-photon entanglement within the error bar of $\sqrt{0.8^2 + 0.4^2}\% = 0.9\%$. This is consistent with the expected negligible crosstalk for the F-qubit, considerably smaller than the theoretical crosstalk for the S-qubit due to spontaneous emission, and far smaller than that due to the resonant global beams.

SUPPLEMENTARY NOTE 3 – EFFECT OF THERMAL PHONON DISTRIBUTION ON QUBIT-TYPE CONVERSION

As shown in Ref. [1], nonzero thermal phonon number can reduce the conversion fidelity between the S-type and F-type qubits, and in particular the performance of the 411 nm laser pulse. (As we can see below, the 3432 nm laser has much longer wavelength so that its effect can be neglected.)

For a given phonon number of n, it is well-known that the carrier Rabi frequency will be modified by a factor of $e^{-\eta^2/2} L_n(\eta^2)$ where η is the Lamb-Dicke parameter, and $L_n(x)$ is the Laguerre polynomial [6]. In this experiment we have $\eta \approx 0.054$ for the 411 nm laser and a trap frequency of $2\pi \times 2.4\,$MHz. For an average phonon number of $\bar{n}$ under thermal distribution, the standard deviation is $\sqrt{\bar{n}(\bar{n}+1)}$, therefore we estimate the fluctuation in the carrier Rabi frequency as $\sqrt{\bar{n}(\bar{n}+1)}\eta^2$ where we use $L_n(\eta^2) \approx 1 - n\eta^2$ when $n\eta^2 \ll 1$. For the qubit-type conversion, we want a π pulse for the 411 nm laser, and the error will be $\cos^2[\frac{\pi}{2}(1 \pm \sqrt{\bar{n}(\bar{n}+1)})] \approx (\pi/2)^2 \bar{n}(\bar{n}+1)\eta^4$. For a round-trip conversion, we have two 411 nm π pulses, so that the error is $2(\pi/2)^2 \bar{n}(\bar{n}+1)\eta^4 \approx 1\%$ for the measured average

phonon number $\bar{n} \approx 16$. This explains about half of the increase in the round-trip conversion fidelity from 1% in our previous work with sideband cooling [1] to 3% here with only Doppler cooling. We suspect that an additional factor of two can appear when we perform two nearby conversions in the spin echo sequence separated barely by a microwave π pulse, such that the pulse area error adds up coherently. If this is the case, we can insert a random phase shift between two adjacent 411 nm conversion pulses in the future to suppress this coherent error.

SUPPLEMENTARY NOTE 4 – CALIBRATION OF ULTRAFAST π PULSE AREA

Figure 1 shows the calibration of the pulse area of the 370 nm pulsed laser.

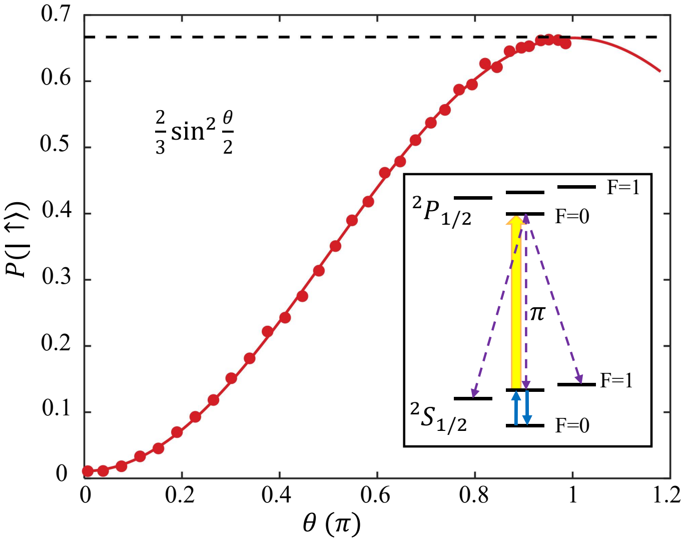

Supplementary Figure 1. **Calibration of the pulse area of 370 nm laser** For a pulse area of θ, the population in the bright state after the spontaneous emission is given by $(2/3)\sin^2(\theta/2)$. We set the laser intensity at the maximum of the curve to obtain a π pulse with an excitation probability of $F_e \approx 99\%$. Inset shows the relevant energy levels.

SUPPLEMENTARY NOTE 5 – MEASUREMENT OF COHERENT QUBIT-TYPE CONVERSION FIDELITY

Figure 2 shows the measurement of the conversion fidelity of the dual-type qubit.

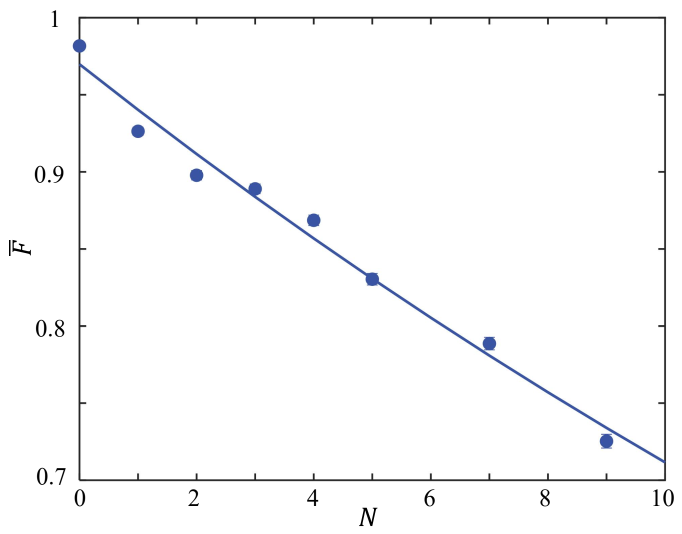

Supplementary Figure 2. **Measurement of conversion fidelity of dual-type qubit.** We repeat the S-F-S round-trip cycles for N times, and fit the average fidelity by $F = F_0(1-\epsilon)^N$ to get the SPAM fidelity of $F_0 = 97.0\%$ and the round-trip conversion error of $\epsilon = 3.1\%$. Each data point is averaged over the six mutually unbiased bases with totally 10^4 samples. The error bar only represents the statistical fluctuation.

[1] H-X Yang, J-Y Ma, Y-K Wu, Ye Wang, M-M Cao, W-X Guo, Y-Y Huang, Lu Feng, Z-C Zhou, and L-M Duan, "Realizing coherently convertible dual-type qubits with the same ion species," Nature Physics **18**, 1058–1061 (2022).

[2] Christopher J Foot, *Atomic physics*, Oxford master series in atomic, optical, and laser physics (Oxford University Press, Oxford, 2007).

[3] R. Yao, W.-Q. Lian, Y.-K. Wu, G.-X. Wang, B.-W. Li, Q.-X. Mei, B.-X. Qi, L. Yao, Z.-C. Zhou, L. He, and L.-M. Duan, "Experimental realization of a multiqubit quantum memory in a 218-ion chain," Phys. Rev. A **106**, 062617 (2022).

[4] Conrad Roman, *Expanding the $^{171}Yb^+$ Toolbox: The $^2F^o_{7/2}$ State as Resource for Quantum Information Science* (University of California, Los Angeles, 2021).

[5] R. Ozeri, W. M. Itano, R. B. Blakestad, J. Britton, J. Chiaverini, J. D. Jost, C. Langer, D. Leibfried, R. Reichle, S. Seidelin, J. H. Wesenberg, and D. J. Wineland, "Errors in trapped-ion quantum gates due to spontaneous photon scattering," Phys. Rev. A **75**, 042329 (2007).

[6] D. Leibfried, R. Blatt, C. Monroe, and D. Wineland, "Quantum dynamics of single trapped ions," Rev. Mod. Phys. **75**, 281–324 (2003).
